# APOA1, DEFB103A_DEFB103B and DSG3 Are Novel Circulating Biomarkers of Psoriasis

**DOI:** 10.3390/ijms27135805

**Published:** 2026-06-26

**Authors:** Monika Dźwigała, Dorota Sys, Joanna Życka-Krzesińska, Beata Rybicka, Piotr Popławski, Irena Walecka-Herniczek, Agnieszka Piekiełko-Witkowska, Joanna Bogusławska

**Affiliations:** 1 Department and Clinic of Dermatology and Paediatric Dermatology, Centre of Postgraduate Medical Education, 02-507 Warsaw, Poland; 2 Department of Translational Immunology and Functional Microbiota Research, Centre of Translational Research, Centre of Postgraduate Medical Education, 01-813 Warsaw, Poland; 3 Department of Biochemistry and Molecular Biology, Centre of Translational Research, Centre of Postgraduate Medical Education, 01-813 Warsaw, Poland

**Keywords:** psoriasis, autoimmunity, cytokines, AGO2, APOA1, DEFB103A, DEFB103B, DSG3, SERPINB4, serum biomarkers

## Abstract

Psoriasis is a chronic inflammatory autoimmune skin disease for which no standardised and reliable molecular biomarkers of disease course or activity are currently available. Here, we aimed to identify serum biomarkers of psoriasis. Serum samples from 40 patients with psoriasis and 40 healthy volunteers were analysed using ELISA and Proximity Extension Assay proteomics. ELISA revealed significantly increased serum levels of AGO2 and APOA1 in psoriatic patients versus controls, with a strong association between APOA1 and psoriasis (OR = 20.72, 95% CI of 4.57–93.87, *p* = 0.000137). Targeted serum proteomics additionally identified 35 differentially expressed proteins, including well-known psoriasis drivers (e.g., top upregulated IL17A and SERPINB4). The most downregulated was adrenomedullin (ADM, FC = −10.12). For 14 altered proteins, no previous direct associations with psoriasis were reported. Among them, DEFB103A_DEFB103B and DSG3 showed the best discrimination between psoriasis and control samples, while SERPINB4 correlated with psoriasis severity. APOA1, DEFB103A_DEFB103B, and DSG3 emerge as novel candidate circulating psoriasis biomarkers, and SERPINB4 as a biomarker of psoriasis severity. The functional role of DSG3 and other newly identified proteins (ACRV1, HAO1, ADH4, GPD1, GFER, PTGES2, DSG3, AFAP1L1, GALNT3, RASGRP2, MAP2K6, LXN, NBEAL2, and VPS54) in psoriasis requires further studies.

## 1. Introduction

Psoriasis (PsO) is a chronic inflammatory autoimmune skin disease characterised by excessive keratinocyte proliferation, their premature differentiation, and the presence of an inflammatory infiltrate in the dermis. The pathogenesis of psoriasis is complex and involves interactions between immunological, genetic, and environmental factors. Particularly important in disease development is the HLA-CW6 allele, as well as external triggers such as stress, infections, mechanical injury, and certain medications. The most common clinical form is plaque psoriasis, which accounts for approximately 85% of all cases [1]. Various clinical scoring systems, such as PASI (Psoriasis Area and Severity Index), BSA (Body Surface Area), and DLQI (Dermatology Life Quality Index), are used to diagnose and assess disease activity [2]. Treatment options include topical and systemic therapies, including biological agents, which are currently regarded as the most effective. It should be emphasised that psoriasis is not merely a skin condition but a chronic, systemic autoimmune inflammatory disease that affects multiple organs and tissues. Scientific evidence indicates that psoriasis significantly increases the risk of psoriatic arthritis, cardiovascular diseases, metabolic disorders (e.g., diabetes), inflammatory bowel disease (IBD), and depression. Unfortunately, no standardised and reliable molecular biomarkers of disease presence, course or activity are currently available [3].

In the search for new biomarkers, increasing attention has been directed towards microRNAs (miRNAs), which play a crucial role in regulating the expression of genes associated with keratinocyte proliferation and immune responses in the skin. Numerous studies have demonstrated that the expression of specific miRNAs is dysregulated in psoriasis, suggesting their potential role as biomarkers of the disease. Their expression in various tissues and presence in body fluids make them promising indicators for early diagnosis, assessment of disease severity, and evaluation of treatment response [4]. It has been shown that analysing the expression of circulating miRNAs (circ-miRNAs) in plasma, serum, or PBMC samples makes it possible to distinguish patients with psoriasis from healthy individuals and to assess therapeutic responses [5]. Despite these promising findings, no miRNA molecule has yet been introduced into routine clinical practice as a biomarker of psoriasis.

MicroRNAs can enter body fluids through active secretion in microvesicles. However, most of the circulating microRNAs are transported not in vesicles but rather in protein complexes [6]. The key protein transporters that carry extracellular microRNAs are Ago proteins (Ago1, AGO2) and HDL lipoproteins [7,8,9]. The majority of circulating microRNAs is transported in complex with AGO2 [6,10]. Recent studies demonstrated that Ago proteins are involved in immune regulation [11,12,13] and inflammation [14,15], while anti-Ago antibodies have been detected in autoimmune disorders [16,17,18,19]. Moreover, it was demonstrated that AGO2 expression in psoriatic skin is reduced, while IL-22, a proinflammatory cytokine and psoriasis-inducing factor, decreases AGO2 in keratinocytes [20]. Ago1 is a transporter of the recently identified autoimmune-related AGO-taxis small RNAs (ASRs). Specifically, ASRs are derived from Y RNAs, which are the major components of autoangiogenic Ro proteins [21]. Recent studies showed that Ago1 expression is altered in rheumatoid arthritis and is a potential therapeutic target in RA [22]. Anti-Ago1 antibodies are detected in a broad spectrum of autoimmune diseases, including Systemic Lupus Erythematosus [17] and Autoimmune Sensory Neuronopathy [23,24]. AGO2 has emerged as an important regulator of immune and inflammatory responses. AGO2-dependent microRNA pathways participate in the regulation of immune cell differentiation, inflammatory signalling, and innate immune responses. In particular, AGO2 has been shown to contribute to monocyte differentiation and lipopolysaccharide-induced inflammatory responses, highlighting its role beyond extracellular microRNA transport [25]. Similarly, APOA1 regulates the activity of monocytes/macrophages, dendritic cells, neutrophils, and T lymphocytes and modulates multiple inflammatory pathways [26]. Therefore, alterations in circulating APOA1 and AGO2 levels may reflect broader immune dysregulation associated with psoriasis rather than isolated molecular changes.

Here, we aimed to identify potential minimally invasive serum biomarkers of psoriasis. To this end, we analysed serum concentrations of AGO1, AGO2, and APOA1 in patients with psoriasis and healthy volunteers. Given the emerging immunomodulatory roles of AGO2 and APOA1, we hypothesised that alterations in their circulating levels may occur in parallel with broader changes in immune-related proteins and pathways characteristic of psoriasis. Therefore, to determine whether the observed changes in AGO2 and APOA1 were associated with a wider psoriasis-related inflammatory signature, we performed targeted serum proteomics using a PEA platform covering approximately 1000 proteins, including more than 500 inflammation- and immunity-related proteins, representing over 95% of immune-response pathways.

## 2. Results

### 2.1. APOA1 and AGO2 Are Upregulated in PsO Serum Samples

**Figure 1:**
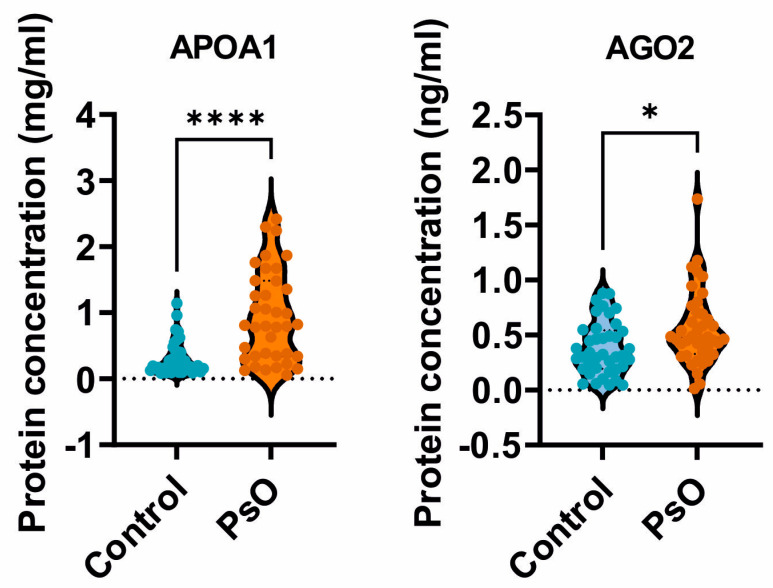
Circulating APOA1 and AGO2 are increased in psoriasis. Serum concentrations of APOA1 and AGO2 in psoriatic (PsO, *n* = 40), and control (Control, *n* = 40) patients. Statistical analysis was done using Mann–Whitney test, * *p* < 0.05, **** *p* < 0.0001.

AGO1 was undetectable with the used ELISA test. In contrast, concentrations of APOA1 and AGO2 were statistically significantly increased in serum samples from psoriatic patients when compared with healthy controls (Figure 1).

There was no correlation between the concentrations of the evaluated proteins and the PASI index or DLQI. However, in the univariable logistic regression analysis, higher serum APOA1 concentration was strongly associated with a significantly increased odds of having the condition (Table 1). Specifically, the odds ratio (OR) for serum APOA1 was 20.72, with a 95% confidence interval (CI) of 4.57–93.87 (*p* = 1.37 × 10^−4^). No such effect was observed for AGO2 (Table 1).

Altogether, this data demonstrated that serum concentrations of APOA1 and AGO2 are increased in psoriatic patients, while elevated APOA1 levels may potentially be a predictor of increased disease risk in this cohort.

### 2.2. Serum Proteomics Reveals Novel PsO Circulating Biomarkers

To place APOA1 and AGO2 in an immune context, PEA omics analysis was conducted to target >90% of immune response REACTOME pathways. This analysis revealed 35 proteins whose expression was altered in PsO serum samples when compared to control samples, including 23 proteins upregulated and 12 with expression decreased (Table 2).

**Figure 2:**
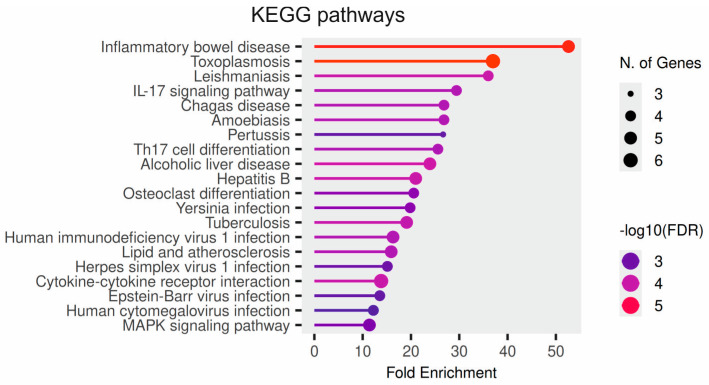
Top enriched KEGG pathways in proteins altered in serum from PsO patients. The plot shows GO analysis of serum proteomics data.

Gene Ontology analysis revealed that the most enriched KEGG pathways included Inflammatory bowel disease, toxoplasmosis, leishmaniasis, and IL-17 signalling pathway (Figure 2).

**Figure 3:**
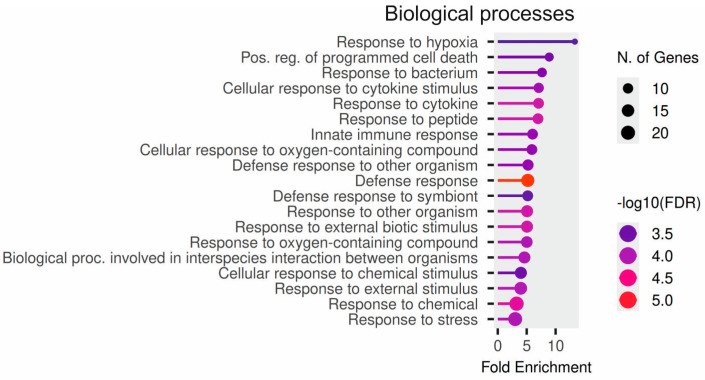
The most enriched biological processes in proteins altered in serum from PsO patients. The plot shows GO analysis of serum proteomics data.

The most enriched biological processes corresponded to response to hypoxia, positive regulation of programmed cell death, response to bacterium, cellular response to cytokine stimulus, as well as response to cytokine, response to peptide, and innate immune response (Figure 3).

**Figure 4:**
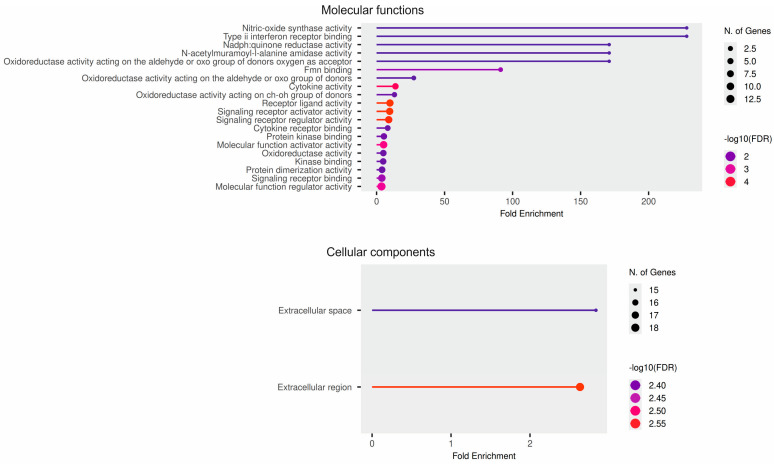
The most enriched molecular functions and cellular components in proteins altered in serum from PsO patients. The plots show GO analysis of serum proteomics data.

The most enriched molecular functions included nitric oxide synthase activity, type II interferon receptor binding, NADPH/quinone reductase activity, and N-acetylmuramoyl-l-alanine amidase activity (Figure 4). In accordance with expectations, the most enriched cellular components were covered by extracellular space and extracellular region (Figure 4).

The most upregulated protein (FC 3.59) was IL17A, while the top decreased was ADM (adrenomedullin, FC −10.12). Remarkably, a substantial fraction (21 out of the 35) of altered proteins was already reported as related either to psoriasis pathology or immune dysregulation (Table 3). These included upregulated IL17A, IL22, IL17C, TNF, IFNG, the key PsO driving cytokines and therapeutic targets [27,28,29,30,31,32,33,34,35,36]. These findings of altered PsO driving cytokines confirmed the robustness of our data. The other upregulated PsO-related proteins included SERPINB4 and GPR15LG, the promoters of keratinocyte inflammation and/or proliferation or biomarkers of PsO severity [37,38,39,40,41,42,43,44,45].

On the other hand, this analysis revealed alterations in other proteins, which were previously not associated with psoriasis. These included upregulated ACRV1 (FC 2.79), HAO1 (FC 2.78), ADH4 (FC 2.09), GPD1 (FC 1.73), GFER (FC 1.61), PTGES2 (FC 1.48), DSG3 (FC 1.40), AFAP1L1 (FC 1.35), and GALNT3 (FC 1.34), as well as downregulated RASGRP2 (FC −2.51), MAP2K6 (FC −2.21), LXN (FC −1.83), NBEAL2 (FC −1.77), and VPS54 (FC −1.68).

### 2.3. DEFB103A_DEFB103B + SERPINB4 Are Potential Serum PsO Diagnostic Biomarkers

**Figure 5:**
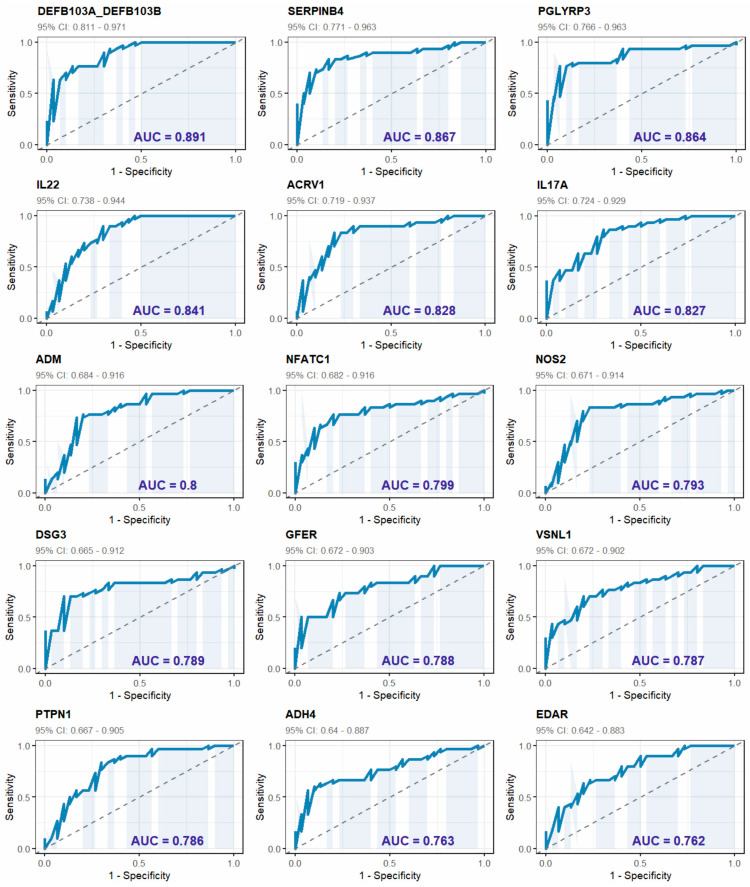
Discriminative performance of serum proteins for psoriasis: individual and cross-validated ROC analyses—Part 1. Individual ROC curves for the top 15 proteins with the highest AUC and 95% CI are shown in each panel subtitle.

**Figure 6:**
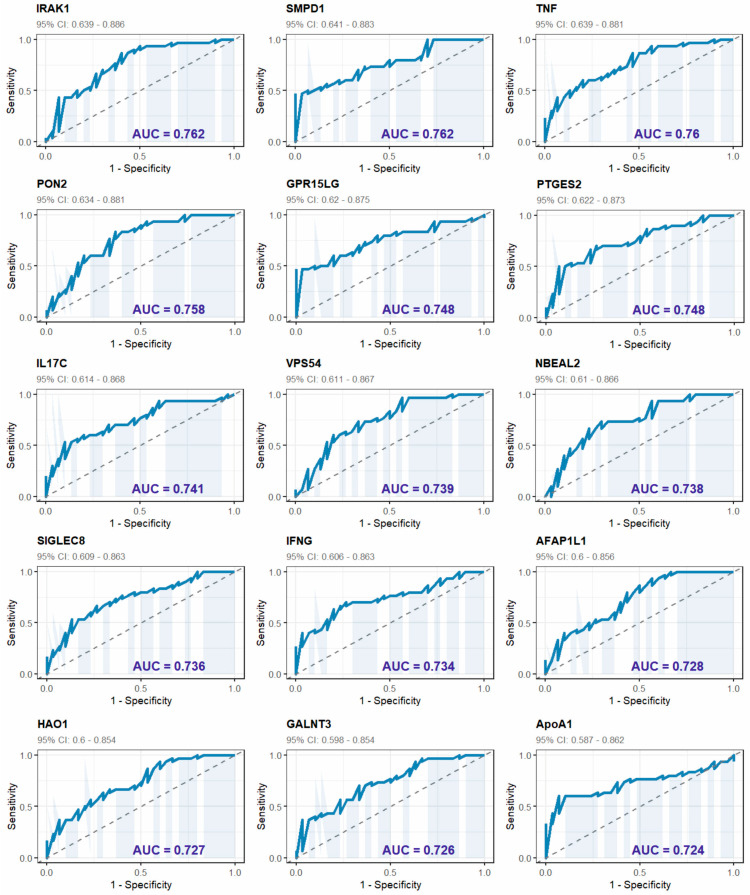
Discriminative performance of serum proteins for psoriasis: individual and cross-validated ROC analyses—Part 2. Individual ROC curves for the remaining 15 proteins whose expression was significantly altered in the serum of psoriatic patients, ordered by AUC (highest first). AUC and 95% CI are shown in each panel subtitle.

**Figure 7:**
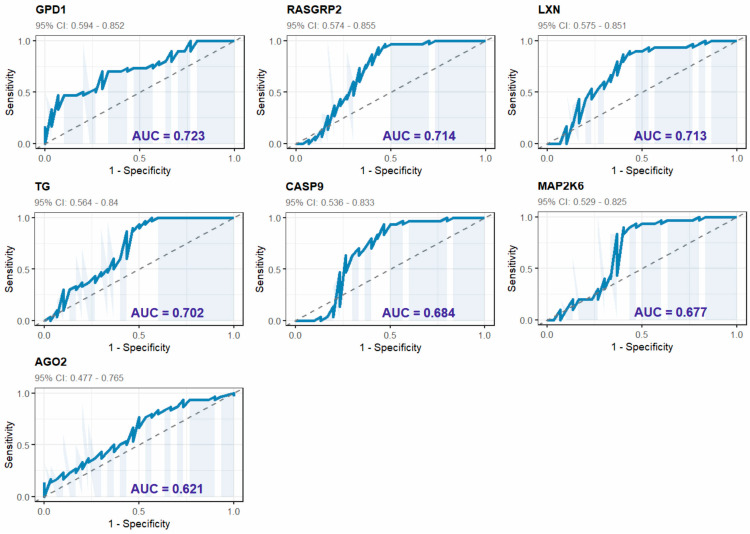
Discriminative performance of serum proteins for psoriasis: individual and cross-validated ROC analyses—Part 3. Individual ROC curves for the remaining 7 proteins whose expression was significantly altered in the serum of psoriatic patients, ordered by AUC (highest first). AUC and 95% CI are shown in each panel subtitle.

To analyse the potential diagnostic value of the identified proteins, each of the statistically significant proteins was evaluated as a binary classifier (Control vs. Psoriasis) using the area under the receiver operating characteristic curve (AUC) (Figure 5, Figure 6 and Figure 7). The direction of the predictor was set automatically. Bootstrap 95% confidence intervals were computed for each AUC estimate. DEFB103A_DEFB103B emerged as the top differentiating protein, followed by SERPINB4 and PGLYRP3 (Figure 5). AUC cross-validation analysis showed consistent performance across folds (Supplementary Data S1).

**Figure 8:**
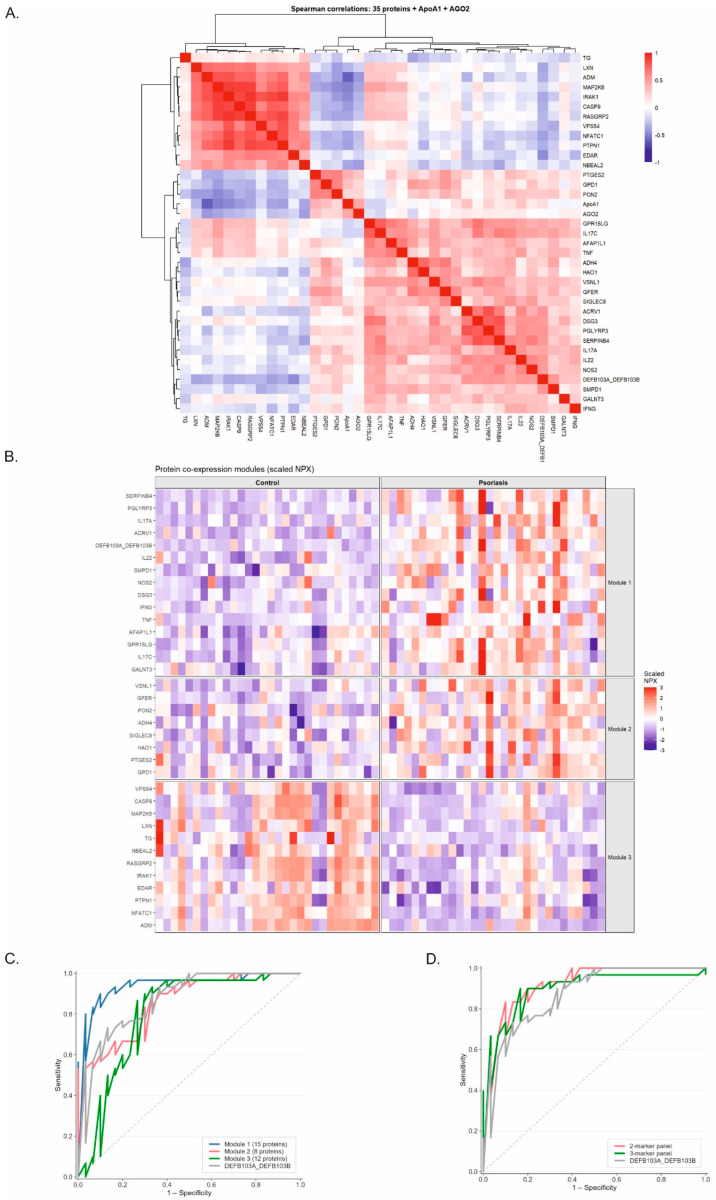
Co-expression patterns and classification performance of protein modules in psoriasis. (**A**) Spearman correlation heatmap with hierarchical clustering (Ward’s D2 linkage). Red indicates positive and blue negative correlations. (**B**) Protein expression heatmap with module and group annotations. Rows represent proteins (ordered by module), and columns represent samples (ordered by group). (**C**) Cross-validated ROC curves for models based on protein co-expression modules and the best single protein. (**D**) Cross-validated ROC curves for models based on reduced protein panels (2- and 3-protein models) and the best single protein. All curves are derived from out-of-fold predictions pooled across 5 repeats of 10-fold cross-validation.

Among module-based models (Table 4), Module 1 achieved the highest discriminative performance, exceeding that of the best single protein (Figure 8C).

To evaluate whether reduced protein sets could maintain predictive performance, we compared the cross-validated AUC (CV-AUC) of the best individual protein, module-based models, and low-dimensional protein panels. While the two-protein (DEFB103A_DEFB103B + SERPINB4) and three-protein (DEFB103A_DEFB103B + SERPINB4 + PGLYRP3) panels showed strong performance, neither outperformed the full Module 1 signature. These findings indicate that although simplified panels retain good discriminative ability, optimal classification is achieved using the broader co-expression module (Table 5, Figure 8D).

**Figure 9:**
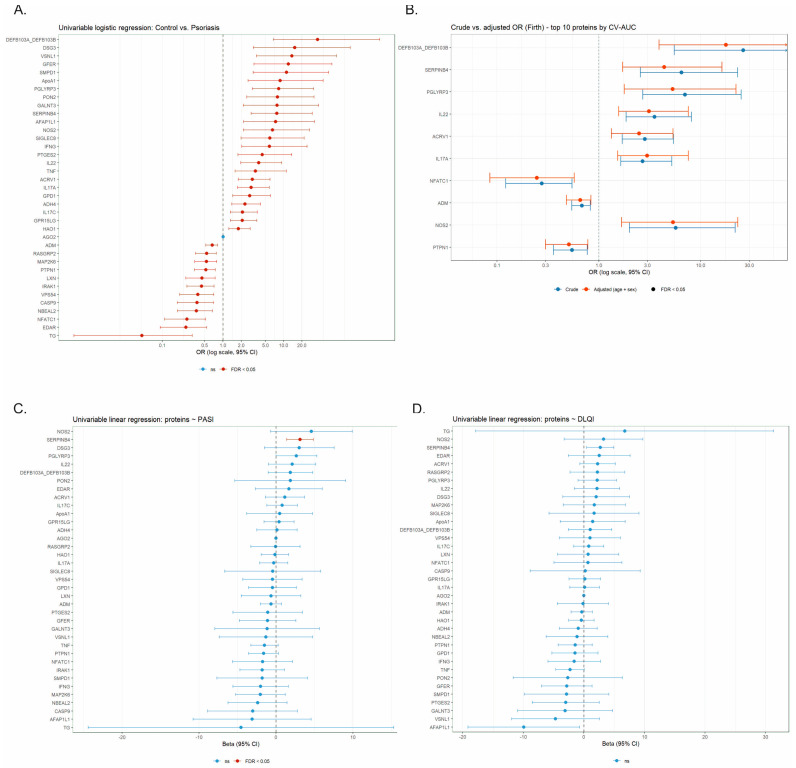
Serum proteins as biomarkers of psoriasis and disease severity. (**A**) Forest plot of univariable logistic regression odds ratios (ORs; log scale). Red indicates FDR < 0.05. (**B**) Forest plot comparing crude and adjusted ORs for the top 10 proteins. Points represent ORs with 95% Wald confidence intervals; red indicates FDR < 0.05. The dashed line denotes OR = 1. (**C**,**D**) Forest plots of univariable linear regression coefficients (β) for PASI (**C**) and DLQI (**D**). Red indicates FDR < 0.05.

To identify the strongest potential individual predictors of psoriasis, proteins were evaluated using univariable logistic regression (Figure 9A). DEFB103A_DEFB103B and DSG3 showed the strongest associations with psoriasis. To assess independence from age and sex, the top 10 proteins (ranked by cross-validated AUC) were further analysed using crude and adjusted logistic regression models (Figure 9B, Supplementary Table S1). Effect estimates remained consistent after adjustment, indicating minimal confounding. DEFB103A_DEFB103B, SERPINB4, PGLYRP3, and NOS2 retained strong positive associations, whereas NFATC1, ADM, and PTPN1 showed inverse associations across both models.

### 2.4. SERPINB4 Is the Best Predictor of PsO Severity

To explore associations with clinical parameters, Spearman’s rank correlations were computed between protein levels and clinical or laboratory variables within the psoriasis group (Supplementary Table S2). The strongest nominal correlations with disease severity were observed for SERPINB4 (PASI: r = 0.474, *p* = 0.011; DLQI: r = 0.534, *p* = 0.006) and TNF (PASI: r = −0.443, *p* = 0.018; DLQI: r = −0.475, *p* = 0.016). However, none of these associations remained significant after FDR correction. To further assess associations with disease severity, proteins were modelled as predictors of PASI and DLQI using univariable linear regression (Figure 9C,D). SERPINB4 showed a significant association with PASI after FDR correction (β = 3.126, 95% CI 1.381–4.871, *p* = 0.001, FDR = 0.039), whereas other proteins did not retain significance. Collectively, these findings identify SERPINB4 as the only protein consistently associated with clinical severity measures across analytical approaches.

## 3. Discussion

In this study, we found that proteins linked with miRNA serum transport, APOA1, and AGO2 are upregulated in psoriatic patients. Furthermore, APOA1 emerged as a potential predictor of risk of disease. In addition, we found numerous other proteins whose concentrations are altered in the serum of psoriatic patients, including upregulated DEFB103A_DEFB103B and DSG3, which emerged as the best potential independent diagnostic PsO biomarkers, as well as SERPINB4, predicted as the best potential biomarker of psoriasis severity.

APOA1 is the major component of HDL, contributing to the 70% of the total protein content of this lipoprotein. It plays a crucial role in the promotion of cholesterol efflux from tissues to the liver [26]. In our study, the odds ratio (OR) for serum APOA1 was 20.72, with a 95% confidence interval (CI) of 4.57–93.87 (*p* = 0.000137). This indicates that, for each unit increase in serum APOA1 (according to the scaling used in the model), the odds of the disease were more than twenty-fold higher, and the confidence interval—although wide—remained entirely above 1.0. The result, therefore, demonstrates a statistically significant and clinically substantial association, suggesting that elevated APOA1 levels may potentially be a predictor of increased disease risk in this cohort. Interestingly, a recent study found that serum concentrations of APOA1 are decreased in patients with psoriatic arthritis when compared with healthy controls as well as patients with rheumatoid arthritis [82]. Based on that data, one could expect that serum APOA1 could potentially serve as a biomarker differentiating between skin PsO and psoriatic arthritis. Unfortunately, the cited report [82] did not involve patients with skin psoriasis; therefore, further studies are needed to analyse whether indeed there are differences in APOA1 concentrations in these two forms of psoriatic disease. Interestingly, APOA1 plays an anti-inflammatory role in endothelial cells and macrophages [26]. This may potentially suggest that increased ApoA1 levels in psoriatic patients may be a form of anti-inflammatory response. In line with this hypothesis, we found negative correlations between APOA1 and MAP2K6, one of the key stress-activated MAPK kinases that phosphorylates and activates p38 MAPK, driving inflammatory and cytokine-responsive transcriptional programmes [83], or IRAK1, a core signal transducer in the MyD88-dependent TLR and IL-1R pathway [84].

We found that AGO2 was upregulated in serum from PsO patients (Figure 1). Although it did not affect the odds of disease, we hypothesise that it may still potentially influence the course of psoriasis as a key molecule that transports miRNAs in the blood [10,13]. Interestingly, our search of the literature revealed that there are at least 15 circulating miRNAs reported as altered in PsO patients [85,86,87], which were also demonstrated as being transported in plasma by AGO2 [10] (Supplementary Table S3). Moreover, AGO2 also protects circulating miRNAs, which are transported in the secreted microvesicles [88], while microvesicle-secreted miRNAs are also associated with PsO pathology [88]. This opens an interesting possibility that altered AGO2 concentrations in the serum of PsO patients could potentially affect the secretion and delivery of miRNA cargo to target cells, including the immune cells. This hypothesis requires further experimental verification. Beyond their potential utility as biomarkers of disease presence and severity, inflammatory pathways associated with psoriasis may also contribute to other clinically relevant consequences of chronic skin inflammation. Post-inflammatory hyperpigmentation (PIH) is increasingly recognised as an important consequence of psoriasis, particularly in individuals with darker skin phototypes. In this context, successful use of Q-switched Nd:YAG laser therapy for residual pigmentation further highlights the broader translational relevance of inflammatory pathways beyond assessment of disease activity alone [89].

DEFB103A_DEFB103B are defensins, the cytotoxic microbicidal peptides produced by neutrophils, well-known for their immunomodulatory properties [90]. Intriguingly, they were already reported as PsO response to treatment biomarkers [51,52], altered expression in PsO skin [53,54], promoters of PsO inflammation [55,56]. To our knowledge, this is the first report of DEFB103A_DEFB103B as a serum PsO biomarker.

SERPINB4 is a well-described mediator of PsO. It has been shown to be overexpressed in PsO skin [37,46] and to promote keratinocyte inflammation. In addition, SERPINB4 has been proposed as a biomarker of skin inflammatory diseases [47] and as a source of autoantigens in inflammatory conditions [38]. Our finding of SERPINB4 as the best potential biomarker of PsO severity is consistent with previous reports [39,40,41,42]. Importantly, among all analysed proteins, SERPINB4 was the only biomarker that remained significantly associated with psoriasis severity after correction for multiple testing. In contrast, most proteins identified in this study demonstrated primarily diagnostic rather than severity-related value, highlighting the distinct biological information provided by disease-presence and disease-severity biomarkers.

Among the 35 serum proteins whose expression was altered in psoriatic patients when compared with controls, we identified fourteen that were not previously reported in the context of psoriasis. These included upregulated DSG3 (Desmoglein 3), a component of desmosome cell–cell junctions, which are required for positive regulation of cellular adhesion [91]. We found that DSG3 was the second-best independent diagnostic PsO biomarker. Although DSG3 exhibited a relatively modest fold change (FC = 1.4), it demonstrated the best independent diagnostic performance in the univariable logistic regression analysis, indicating that discriminatory capacity is not solely determined by the magnitude of differential expression. Fold change reflects differences in group means but does not account for within-group variability or the degree of overlap between distributions. In contrast, logistic regression evaluates the ability of a biomarker to distinguish individual samples across the full data distribution. DSG3 showed relatively low intra-group variability together with a consistent shift between groups, which translated into improved separation and predictive performance despite the modest FC. Although there are no studies linking DSG3 with psoriasis, there are indeed findings that support the hypothesis of its involvement in skin autoimmunity. In particular, DSG3 is required for adherens- and desmosome junction assembly in response to mechanical force in keratinocytes [91], while DSG3 autoantibodies are found in pemphigus vulgaris, an autoimmune blistering disease resulting from the adhesion loss between keratinocytes [92]. The potential involvement of DSG3 in the pathology of psoriasis requires further exploration.

The other upregulated proteins, which could possibly be functionally involved in psoriasis pathology, included HAO1 (Hydroxyacid Oxidase 1), PTGES2 (Prostaglandin E Synthase 2), DSG3 (Desmoglein 3), and AFAP1L (Actin Filament Associated Protein 1-Like 1). HAO1 is expressed primarily in the liver and peroxisomes. Its main function is to oxidise small hydroxy-acids, especially glycolate, producing glyoxylate and hydrogen peroxide [93]. It is known for its impact on inflammation since it negatively regulates macrophage activation in liver disease [94] and activates the formation of neutrophil extracellular traps in lung cancer [95], while neutrophil extracellular traps promote inflammation in PsO [96,97]. The significance of the HAO1 upregulation in the serum of psoriatic patients requires further exploration. PTGES2 (Prostaglandin E Synthase 2), which converts prostaglandin H2 to prostaglandin E2. Interestingly, it was suggested that PGE2 could be involved in the pathogenesis of autoimmune diseases, including psoriasis, by facilitating the expansion of Th17 cells [98]. AFAP1L1 is a relatively weakly recognised protein (only 21 papers reported by PubMed accessed on 30 March 2026). It is mainly associated with cancer progression [99,100,101,102,103,104,105,106,107,108,109,110,111]. The sequence variants of the AFAP1L1 gene are associated with severe diabetic retinopathy in type 1 diabetes [112]. Interestingly, two papers suggest the association of AFAP1L1 with immune processes: it was demonstrated that AFAP1L1 expression is altered in patients allergic to grass pollen when compared with healthy controls [113], while in clear cell renal cell carcinoma, AFAP1L1 was reported as an immune-related biomarker, correlating with the presence of neutrophils and macrophages [99]. The exact functional relationship between AFAP1L1 and autoimmune disorders and psoriasis requires further exploration.

We also detected increased serum expression of proteins for which there are no functional links with inflammation or autoimmunity. These included ACRV1 (Acrosomal Vesicle Protein 1), ADH4 (Alcohol Dehydrogenase 4 (Class II)), GALNT3 (Polypeptide N-Acetylgalactosaminyltransferase 3), GPD1 (Glycerol-3-Phosphate Dehydrogenase 1), and GFER (Growth Factor, Augmenter of Liver Regeneration). Of them, the most upregulated (FC 2.79) was ACRV1, whose expression is physiologically restricted to male germ cells [114]. However, recent studies showed that ACRV1 expression is pathologically reactivated in non-testis tissues, including ovarian cancer and other cancer types [115]. ARCV1 mRNA was also reported as a saliva biomarker of pancreatic cancer [116]. Further studies are required to explore the significance of ACRV1, ADH4, and GALNT3 upregulation in psoriatic serum samples.

We observed increased expressions of GFER and GPD1 in serum from psoriatic patients. GFER is a multifunctional protein that acts as an FAD-linked sulfhydryl oxidase and cytochrome c reductase. It is also involved in the regulation of lipid homeostasis and inflammation [117,118]. Its serum levels are decreased in diabetic nephropathy [119], Nonalcoholic Steatohepatitis and Fibrosis [120], while being increased in inflammation [121], acute-on-chronic liver failure, and hepatocarcinoma [122]. Remarkably, GFER is a powerful immunoregulator. In a murine model of pancreatic adenocarcinoma, GFER depletion stimulates T-cell infiltration and attenuates tumour growth [123]. In contrast, in the rat model of renal ischemia/reperfusion injury, GFER decreases neutrophil and macrophage infiltration in the tubulointerstitial, leading to a decrease in inflammatory cytokines [124], and inhibits apoptosis of activated peripheral lymphocytes [125]. GPD1 catalyses the conversion of dihydroxyacetone phosphate (DHAP) to glycerol 3-phosphate in the cytoplasm. It is also associated with numerous disorders, including obesity, transient infantile hypertriglyceridemia, and neuroinflammation [126]. There are no published studies directly linking GFER and GDP1 with autoimmune skin diseases. However, they both are associated with liver damage [117,127]. Moreover, we observed moderate correlations between AST and GPD1 or GFER in psoriatic patients. Given that psoriasis is increasingly recognised as a systemic disease associated with hepatic dysfunction [128,129,130], this may potentially suggest that circulating GDP1 and GFER levels could reflect hepatocellular stress in psoriatic patients.

The most downregulated protein in the serum of psoriatic patients was adrenomedullin (ADM) (FC −10.12). Koczan et al. [81] reported that ADM expression in PBMC of severe generalised psoriasis patients was moderately upregulated (FC 1.69, *p* = 0.0479) when compared to PBMC from the same patients after effective treatment. However, that study was conducted on a very small group of patients (*n* = 11) and did not involve healthy controls; it is difficult to conclude on the clinical significance of those findings. ADM is a peptide produced mainly by adrenal glands with a broad scope of activity, encompassing vasodilation, inhibition of aldosterone and ACTH secretion, as well as antioxidative, antifibrotic, angiogenic, antimicrobial, and immunomodulatory activities [131]. Interestingly, ADM is endogenously expressed by keratinocytes [132] and stimulates their cellular growth and proliferation while inhibiting apoptosis [133]. ADM is also an antimicrobial peptide and contributes to the mucosal host defence [134]. Intriguingly, it was suggested that ADM plays a protective role in autoimmune disease by reducing NFkB signalling and IL6 secretion [135]. Specifically, ADM plays a protective role in autoimmune encephalomyelitis [136], multiple sclerosis and rheumatoid arthritis [137], and autoimmune uveitis [138]. Our study suggests that a similar protective role could be played by ADM in psoriasis, and the loss of ADM could possibly contribute to the course of the disease.

The second protein most decreased in the serum of psoriatic patients was RASGRP2 (RAS guanyl nucleotide-releasing protein 2). It belongs to the family of guanine nucleotide exchange factors, which activate small GTPases and thereby contribute to the key signalling pathways. RASGRP2 is best known for its role in platelets, where it activates pathways linked with integrins and calcium, thereby contributing to platelet aggregation and thrombosis. It is also expressed by neutrophils and T cells, as well as endothelium, fibroblast-like synoviocytes, and the brain, where it participates in dopamine-dependent signalling. RASGRP2 mutations are associated with bleeding disorders [139]. To the best of our knowledge, there are no published studies showing RASGRP2 associations with psoriasis. However, it is linked with autoimmunity. Specifically, it acts as an autoantigen in multiple sclerosis [140]. In rheumatoid arthritis, RASGRP2 promotes adhesion, migration, and production of IL-6 in fibroblast-like synoviocytes, thereby contributing to the development of destructive arthritis [141]. Moreover, RASGRP2 contributes to immune-mediated thrombocytopenia and thrombosis syndromes (ITT) [142] and possibly to systemic lupus erythematosus [143]. Such a broad autoimmune context suggests that RASGRP2 could also be involved in the pathogenesis of psoriasis. Supportive of this view is the finding that in psoriasis, platelets undergo disease-specific immune reprogramming [144], are activated [145], and contribute to the inflammation in psoriatic skin by releasing several cytokines [146,147], while RASGRP2 is abundantly expressed in platelets and directly contributes to their activation [139]. Interestingly, we found strong correlations between RASGRP2 and other platelet- or psoriasis-related proteins (e.g., MAP2K6, IRAK1, or PTPN1) (Figure 8A). The specific role of RASGRP2 in psoriasis requires further exploration.

The other downregulated proteins, for which no previous studies reported associations with autoimmune skin disease, included LXN (Latexin), NBEAL2 (Neurobeachin Like 2), and VPS54 (Vacuolar Protein Sorting-Associated Protein 54). Interestingly, there are studies that indirectly suggest their potential associations with psoriasis. For instance, it was shown that LXN deficiency stimulates inflammation in a murine model of colitis [148], while NBEAL2 is required for NK and neutrophils [149] and mast cells [150], and its deficiency leads to activation of T cells [151]. VPS54 is an essential component of the GARP complex that is involved in retrograde transport from early and late endosomes to the trans-Golgi network (TGN) [152]. Interestingly, psoriasis is partially driven by endosomal activation of TLR7/8/9 receptors, which in turn stimulates type I interferons, neutrophil activation, and inflammation [153]. It could be, therefore, hypothesised that the GARP complex, together with VSP54, could influence the localisation, recycling, or degradation of TLRs, thereby contributing to the psoriasis pathology. This hypothesis, as well as the significance of lowered serum VPS54 in psoriasis, requires further study.

This study has several limitations which must be acknowledged. First, the sample size was small, particularly considering the high-dimensional proteomic data set generated by the PEA platform. However, to reduce the possibility of false positive results and overfitting of the model, we performed False Discovery Rate correction and repeated cross-validation procedures, but some overestimation of the performance cannot be entirely excluded. Thus, the diagnostic performance of the identified biomarkers should be taken with caution. Second, the study was performed on a single cohort and lacked independent external validation. Therefore, the identified proteins should be considered as promising candidate biomarkers, but their diagnostic utility needs to be confirmed in larger, independent patient populations before a potential clinical application can be established. Third, there are a number of clinical factors that may influence circulating protein concentrations, such as treatment status, disease duration, smoking, metabolic comorbidities, and BMI, that could potentially influence the observed associations. Clinical characteristics are available (Supplementary Table S4), but the present study was not powered to perform stratified analyses according to these variables. Moreover, it was not possible to adjust for BMI in the analyses because BMI data were not available for the control group. Thus, the potential influence of these factors on the identified biomarkers cannot be ruled out and should be investigated in future studies in larger and clinically well-characterised cohorts. Finally, the cross-sectional design of the study does not allow concluding causal relationships between the identified proteins and the pathogenesis of psoriasis. Longer longitudinal, mechanistic and multicentre studies are needed to establish whether these proteins have a direct biological role in disease development or are predominantly biomarkers associated with disease presence and severity. In conclusion, we report that serum concentrations of miRNA transport-related proteins APOA1 and AGO2 are elevated in psoriatic patients, while APOA1 represents a potential predictor of risk of the disease. In addition, APOA1, DEFB103A_DEFB103B and DSG3 emerge as promising candidate circulating biomarkers of psoriasis, while SERPINB4 may represent a biomarker of disease severity. However, these findings require validation in larger independent cohorts before their potential clinical utility can be established. The functional role of altered serum levels of DSG3 as well other newly identified proteins (ACRV1, HAO1, ADH4, GPD1, GFER, PTGES2, DSG3, AFAP1L1, GALNT3, RASGRP2, MAP2K6, LXN, NBEAL2, and VPS54) in psoriasis remains to be elucidated. Beyond their diagnostic value, the identified biomarkers may also have potential translational relevance. In particular, severity-associated proteins such as SERPINB4 could contribute to monitoring disease burden and treatment response. Furthermore, several dysregulated proteins identified in the PEA analysis were linked to inflammatory pathways already known to play central roles in psoriasis pathogenesis, including TNF-, IL-17-, and IL-22-associated signalling networks. Although the present study was not designed to identify therapeutic targets, these findings provide additional insight into the molecular landscape of psoriasis and may help prioritise pathways and candidate molecules for future mechanistic and therapeutic investigations.

## 4. Materials and Methods

### 4.1. Study Population and Serum Collection

Serum samples were collected from 40 patients with psoriasis (PsO) and 40 healthy volunteers (Control) at the Department and Clinic of Dermatology and Paediatric Dermatology of the Centre of Postgraduate Medical Education. All participants provided written informed consent, and the study was conducted under approval of the local Bioethics Committee (approval no. 111/PB/2020). Venous blood was collected into serum tubes, allowed to clot and centrifuged according to standard procedures. Serum aliquots were stored at −80 °C until analysis. Haemolysis was evaluated spectrophotometrically at 414 nm. All samples with absorbance >0.2, indicative of relevant haemolysis, were excluded from further analysis.

### 4.2. ELISA Measurements

Serum concentrations of AGO1, AGO2, and APOA1 proteins were evaluated using a Human Protein Argonaute-2 (AGO2) ELISA Kit (cat. no. abx515447, Abbexa, Cambridge, UK), a Human Protein Argonaute 1/EIF2C1 (AGO1) ELISA Kit (cat. no. abx387088, Abbexa), a Human APOA1 ELISA Kit (cat. no. KE00157, Proteintech, Rosemont, IL, USA), respectively. Analysis was conducted in accordance with the manufacturer’s instructions. Samples were analysed in duplicate, and mean values were used for analysis. Values below the limit of detection (LoD) were set to zero. AGO1 levels were below LoD in the majority of samples and were therefore excluded from further analysis.

### 4.3. Serum Proteomics (Olink PEA)

Proteomic analysis was performed in a subset of 30 PsO samples and 30 control samples using the Olink^®^ Proximity Extension Assay (PEA) technology and processed via the Olink^®^ Reveal, which targets 1034 proteins, including 537 linked to inflammation, and covers 96% of immune-response pathways in Reactome, all of its top-level pathways, and 64% of all Reactome pathways. Analysis was conducted by Novogene following the manufacturer’s recommendations. Briefly, samples were incubated with pairs of oligonucleotide-labelled antibodies specific to target proteins; upon dual binding, the oligonucleotides hybridised and were extended by PCR, generating assay- and sample-specific barcoded amplicons that were pooled, purified using magnetic beads, and sequenced on a NovaSeq 6000 (Illumina, San Diego, CA, USA) platform. Sequencing reads were converted to counts and normalised to NPX (Normalised Protein eXpression) values on a log_2_ scale, where higher NPX reflects higher protein abundance. Differential protein expression was assessed using standardised NPX values with *t*-tests or ANOVA, as appropriate, implemented via the OlinkAnalyze R package version 3.5.1, and significance was determined based on adjusted *p*-values.

### 4.4. Statistical and Bioinformatic Analysis

Statistical analysis of ELISA data was performed using GraphPad Prism 10. GO analysis was done using ShinyGO 0.85 [154]. Proteomic data analysis was performed in R (version 4.5.1). Protein concentrations are expressed in NPX (Normalised Protein eXpression) units on a log_2_ scale, as provided by the Olink platform.

Differential expression between psoriasis patients and healthy controls was assessed using the Olink differential expression pipeline. The discriminatory ability of each protein was quantified by the area under the receiver operating characteristic curve (AUC) with bootstrap 95% confidence intervals (pROC package ver. 1.19.0.1). To account for overfitting, cross-validated AUC (CV-AUC) was estimated using 100-repeat 5-fold cross-validation; optimism was defined as raw AUC—CV-AUC.

Co-expression modules were identified by hierarchical clustering (Ward’s D2 linkage) applied to a Spearman rank correlation matrix of the 35 statistically significant proteins; the number of modules was set to k = 3, chosen based on dendrogram structure and interpretability. Module scores were computed as the mean of standardised (z-scored) NPX values within each module and compared between groups using the Mann–Whitney U test with BH-FDR correction. Associations between module scores and disease severity indices (PASI, DLQI) were assessed by Spearman’s rank correlation. The discriminative performance of module scores and reduced protein panels (2- and 3-protein combinations selected from top-ranked proteins) was evaluated by CV-AUC estimated from out-of-fold predictions pooled across 5 repeats of 10-fold cross-validation, using simple logistic regression as the base classifier. A different cross-validation scheme was intentionally used for multivariable models to maximise training data within each fold.

To assess potential confounding by age and sex, the top 10 proteins ranked by CV-AUC were analysed using crude and adjusted logistic regression. Firth’s penalised logistic regression (logistf package ver. 1.26.1) was applied to handle quasi-complete separation common in small proteomic studies. Results are expressed as odds ratios (OR) with profile-likelihood 95% confidence intervals; BH-FDR correction was applied per model. BMI adjustment was not feasible as BMI data was unavailable for control participants.

Associations between protein levels and clinical or laboratory variables (PASI, DLQI, BSA, age, BMI, ALT, AST, CRP, ESR, creatinine) within the psoriasis group were assessed by Spearman’s rank correlation with BH-FDR correction applied per outcome. Proteins were additionally modelled as predictors of PASI and DLQI using univariable linear regression; results are expressed as regression coefficients (β) with 95% confidence intervals. Statistical significance was defined as adjusted *p*-value (BH-FDR) < 0.05 unless stated otherwise.

## Figures and Tables

**Table 1:** Results of univariable logistic regression for circulating APOA1 and AGO2 differentiating PsO and Control groups.

Variable	OR (e^β)	Lower 95% CI	Upper 95% CI	*p*-Value
APOA1	20.72	4.57	93.87	1.37 × 10^−4^
AGO2	1.00	1.00	1.00	1.36 × 10^−2^

**Table 2:** Serum proteins were altered in PsO samples when compared to control samples. The table shows results of PEA omics analysis, FC threshold |1.3|.

Protein Name	UniProt ID	FC	*p*-Value	Adjusted_Pval
IL17A	Q16552	3.59	9.01 × 10^−6^	2.33 × 10^−3^
SERPINB4	P48594	2.89	3.99 × 10^−6^	2.33 × 10^−3^
ACRV1	P26436	2.79	7.07 × 10^−6^	2.33 × 10^−3^
HAO1	Q9UJM8	2.78	0.001193	0.042328231
IL22	Q9GZX6	2.29	1.41 × 10^−5^	2.60 × 10^−3^
GPR15LG	Q6UWK7	2.28	0.001123	0.042328231
PGLYRP3	Q96LB9	2.22	5.51 × 10^−6^	2.33 × 10^−3^
IL17C	Q9P0M4	2.22	0.001321	0.04405857
TNF	P01375	2.19	0.001219	0.042328231
ADH4	P08319	2.09	0.000719	0.037801669
DEFB103A_DEFB103B	P81534	2.00	1.99 × 10^−5^	2.94 × 10^−3^
GPD1	P21695	1.73	0.001599	0.044427137
GFER	P55789	1.61	0.000284	0.020972116
IFNG	P01579	1.54	0.000768	0.037801669
PTGES2	Q9H7Z7	1.48	0.001228	0.042328231
SMPD1	P17405	1.45	6.95 × 10^−5^	0.007182787
NOS2	P35228	1.44	0.000483	0.031189917
VSNL1	P62760	1.44	4.41 × 10^−5^	5.70 × 10^−3^
DSG3	P32926	1.40	0.000746	0.037801669
PON2	Q15165	1.39	0.000311	0.021463862
SIGLEC8	Q9NYZ4	1.36	0.00112	0.042328231
AFAP1L1	Q8TED9	1.35	0.000942	0.038941053
GALNT3	Q14435	1.34	0.001499	0.044427137
TG	P01266	−1.30	0.001895	0.048978471
EDAR	Q9UNE0	−1.67	0.000256	0.02032358
VPS54	Q9P1Q0	−1.68	0.001619	0.044427137
CASP9	P55211	−1.68	0.001886	0.048978471
NBEAL2	Q6ZNJ1	−1.77	0.000864	0.038832519
NFATC1	O95644	−1.83	5.56 × 10^−5^	6.38 × 10^−3^
LXN	Q9BS40	−1.99	0.001633	0.044427137
IRAK1	P51617	−2.11	0.000536	0.032627577
MAP2K6	P52564	−2.21	0.001617	0.044427137
RASGRP2	Q7LDG7	−2.51	0.000829	0.038832519
PTPN1	P18031	−3.00	0.000212	0.018301167
ADM	P35318	−10.12	1.51 × 10^−5^	2.60 × 10^−3^

**Table 3:** The functions of serum proteins were altered in PsO samples when compared to control samples.

Protein Name	UniProt ID	Links with Psoriasis
IL17A	Q16552	One of the key PsO drivers; activates keratinocyte proliferation; a target for therapy [27,28,29,30]
SERPINB4	P48594	Promotes keratinocyte inflammation, overexpressed in PsO skin [37,46], biomarker of skin inflammatory diseases [47], a source of autoantigen in inflammatory diseases [38], correlates with PsO severity [39,40,41,42]
ACRV1	P26436	No direct links
HAO1	Q9UJM8	No direct links
IL22	Q9GZX6	One of the key PsO drivers; potential therapeutic target, biomarker of response to treatment [31,32,33,34]
GPR15LG	Q6UWK7	Alias C10orf99; contributes to PsO development in animal model [43]; promotes keratinocyte proliferation in PsO [44], regulates proinflammatory response in PsO [45]
PGLYRP3	Q96LB9	SNPs linked with PsO [48,49]; However: proved not to be involved in development of PsO in mouse model [50]
IL17C	Q9P0M4	One of the key PsO drivers [28]
TNF	P01375	One of the key PsO drivers and therapeutic targets [35]
ADH4	P08319	No direct links
DEFB103A_DEFB103B	P81534	Alias hBD-3, defensin, antimicrobial activity, PsO biomarker of response to treatment [51,52], altered expression in PsO skin [53]; upregulated in PsO lesions [54], promotes PsO inflammation [55]; increases expression of IL-37 in keratinocytes [56]
GPD1	P21695	No direct links
GFER	P55789	No direct links
IFNG	P01579	One of the key PsO drivers [36]
PTGES2	Q9H7Z7	No direct links
SMPD1	P17405	Sphingomyelinase, which breaks down sphingomyelin into ceramide, a central sphingolipid molecule; ceramide metabolism is altered in psoriatic skin [57]; downregulated in keratinocytes in response to retinoids [58]; upregulated in psoriatic skin lesions [59]; upregulated by tapinarof, an AHR ligand approved for the treatment of psoriasis [60]
NOS2	P35228	Driver of PsO [61,62,63]
VSNL1	P62760	[64]
DSG3	P32926	No direct links
PON2	Q15165	Paraoxonase 2; no changes in PsO [65]
SIGLEC8	Q9NYZ4	Altered in PsO [66]
AFAP1L1	Q8TED9	No direct links
GALNT3	Q14435	No direct links
TG	P01266	Anti-Tg antibodies present in PsO patients [67]
EDAR	Q9UNE0	Involved in PsO [68] and epidermal homeostasis [69]
VPS54	Q9P1Q0	No direct links
CASP9	P55211	Decrease in serum of psoriatic arthritis patients [70]
NBEAL2	Q6ZNJ1	No direct links
NFATC1	O95644	NFATc1 supports imiquimod-induced skin inflammation [71]
LXN	Q9BS40	No direct links
IRAK1	P51617	Involved in PsO [72,73,74,75]
MAP2K6	P52564	Involved in keratinocyte proliferation and migration [76,77]
RASGRP2	Q7LDG7	No direct links
PTPN1	P18031	Decreased in peripheral white blood cells of patients with psoriatic type 2 diabetes when compared with healthy controls [78]; a potential susceptibility gene for psoriasis [79], upregulated in skin lesions of psoriatic patients when compared with non-affected skin [80]
ADM	P35318	Altered in PBMC in response to treatment, potentially involved in PsO [81]

**Table 4:** Protein co-expression module composition (Ward’s D2 hierarchical clustering, k = 3).

Module	N Proteins	Proteins
Module 1	15	ACRV1, AFAP1L1, DEFB103A_DEFB103B, DSG3, GALNT3, GPR15LG, IFNG, IL17A, IL17C, IL22, NOS2, PGLYRP3, SERPINB4, SMPD1, TNF
Module 2	8	ADH4, GFER, GPD1, HAO1, PON2, PTGES2, SIGLEC8, VSNL1
Module 3	12	ADM, CASP9, EDAR, IRAK1, LXN, MAP2K6, NBEAL2, NFATC1, PTPN1, RASGRP2, TG, VPS54

**Table 5:** Comparison of cross-validated discriminative performance (CV-AUC) across single proteins, co-expression modules, and reduced protein panels.

Panel	CV-AUC	Δ vs. Single
Module 1 (15 proteins)	0.947	0.056
2 proteins (DEFB103A_DEFB103B + SERPINB4)	0.925	0.035
3 proteins (DEFB103A_DEFB103B + SERPINB4 + PGLYRP3)	0.907	0.017
1 protein (DEFB103A_DEFB103B)	0.891	0.000
Module 2 (8 proteins)	0.856	−0.035
Module 3 (12 proteins)	0.801	−0.090

## Data Availability

The original contributions presented in this study are included in the article/Supplementary Materials. Further inquiries can be directed to the corresponding authors.

## Supplementary Materials

The following supporting information can be downloaded at: https://www.mdpi.com/article/10.3390/ijms27135805/s1.

## Abbreviations

The following abbreviations are used in this manuscript:

ADM	adrenomedullin
AFAP1L1	Actin Filament-Associated Protein 1-Like 1
AGO1	Argonaute 1
AGO2	Argonaute 2
ALT	alanine aminotransferase
AST	aspartate aminotransferase
AUC	area under the curve
BSA	Body Surface Area
CI	confidence interval
CRP	C-reactive protein
CV-AUC	cross-validated area under the curve
DEFB103A_DEFB103B	defensin beta 103A/103B
DLQI	Dermatology Life Quality Index
DSG3	desmoglein 3
ELISA	enzyme-linked immunosorbent assay
ESR	erythrocyte sedimentation rate
FC	fold change
FDR	False Discovery Rate
GO	Gene Ontology
HAO1	hydroxyacid oxidase 1
HDL	high-density lipoprotein
IBD	inflammatory bowel disease
IFNG	interferon gamma
IJMS	*International Journal of Molecular Sciences*
IL	interleukin
LoD	limit of detection
LXN	latexin
MAP2K6	mitogen-activated protein kinase kinase 6
miRNA	microRNA
NPX	Normalised Protein eXpression
OR	odds ratio
PASI	Psoriasis Area and Severity Index
PBMC	peripheral blood mononuclear cell
PBMCs	peripheral blood mononuclear cells
PEA	Proximity Extension Assay
PsA	psoriatic arthritis
PsO	psoriasis
PTGES2	prostaglandin E synthase 2
QC	quality control
RA	rheumatoid arthritis
ROC	receiver operating characteristic
SLE	systemic lupus erythematosus
SMPD1	sphingomyelin phosphodiesterase 1
TNF	tumour necrosis factor
