# Supplementary material for: APOA1, DEFB103A_DEFB103B and DSG3 Are Novel Circulating Biomarkers of Psoriasis
Source: Int J Mol Sci. 2026 Jun 26;27(13):5805. doi: 10.3390/ijms27135805 (PMC13362322; doi:10.3390/ijms27135805)
Supplement: Supplementary file 1 [file ijms-27-05805-s001.zip › Supplementary Data S1_corrected.pdf]

Supplementary Data S1. Cross-validation of AUC (repeated 5-fold CV).

Raw AUC is an optimistic in-sample estimate. To quantify optimism, each analyte was also evaluated using 100-repeat 5-fold cross-validation: the data were split 100 times into 5 folds; each fold was held out once as test set. This gave a distribution of CV-AUC values whose mean approximates the expected out-of-sample performance.

Raw vs. cross-validated AUC (100 × 5-fold CV) for all 37 analytes. Optimism = Raw AUC – CV AUC.

Protein	Raw AUC	CV AUC (95% CI)	Optimism
DEFB103A_DEFB103B	0.891	0.893 (0.704–1.082)	-0.002
SERPINB4	0.867	0.866 (0.665–1.068)	0
PGLYRP3	0.864	0.864 (0.654–1.075)	0
IL22	0.841	0.839 (0.606–1.073)	0.002
ACRV1	0.828	0.828 (0.596–1.061)	0
IL17A	0.827	0.828 (0.594–1.062)	-0.001
NFATC1	0.799	0.809 (0.565–1.054)	-0.011
ADM	0.800	0.799 (0.558–1.04)	0.001
NOS2	0.793	0.794 (0.538–1.05)	-0.001
PTPN1	0.786	0.794 (0.543–1.044)	-0.007
DSG3	0.789	0.788 (0.535–1.041)	0.001
GFER	0.788	0.787 (0.524–1.05)	0.001
VSNL1	0.787	0.787 (0.541–1.032)	0
PON2	0.758	0.765 (0.529–1.001)	-0.007
ADH4	0.763	0.764 (0.499–1.03)	-0.001

Protein	Raw AUC	CV AUC (95% CI)	Optimism
TNF	0.760	0.762 (0.507–1.016)	-0.002
EDAR	0.762	0.761 (0.498–1.025)	0.001
IRAK1	0.762	0.761 (0.491–1.032)	0.001
SMPD1	0.762	0.758 (0.495–1.021)	0.004
GPR15LG	0.748	0.755 (0.491–1.019)	-0.008
PTGES2	0.748	0.752 (0.49–1.014)	-0.005
IL17C	0.741	0.747 (0.481–1.014)	-0.006
SIGLEC8	0.736	0.746 (0.508–0.985)	-0.01
IFNG	0.734	0.745 (0.488–1.001)	-0.01
VPS54	0.739	0.741 (0.481–1.002)	-0.002
NBEAL2	0.738	0.74 (0.471–1.009)	-0.002
ApoA1	0.724	0.734 (0.457–1.011)	-0.01
RASGRP2	0.714	0.732 (0.476–0.987)	-0.017
HAO1	0.727	0.73 (0.454–1.005)	-0.003
GPD1	0.723	0.727 (0.459–0.994)	-0.003
GALNT3	0.726	0.725 (0.44–1.009)	0.001
LXN	0.713	0.725 (0.473–0.978)	-0.012
AFAP1L1	0.728	0.722 (0.434–1.01)	0.005
TG	0.702	0.72 (0.452–0.989)	-0.018

Protein	Raw AUC	CV AUC (95% CI)	Optimism
CASP9	0.684	0.702 (0.43–0.975)	-0.018
MAP2K6	0.677	0.698 (0.438–0.958)	-0.021
AGO2	0.621	0.657 (0.402–0.912)	-0.036

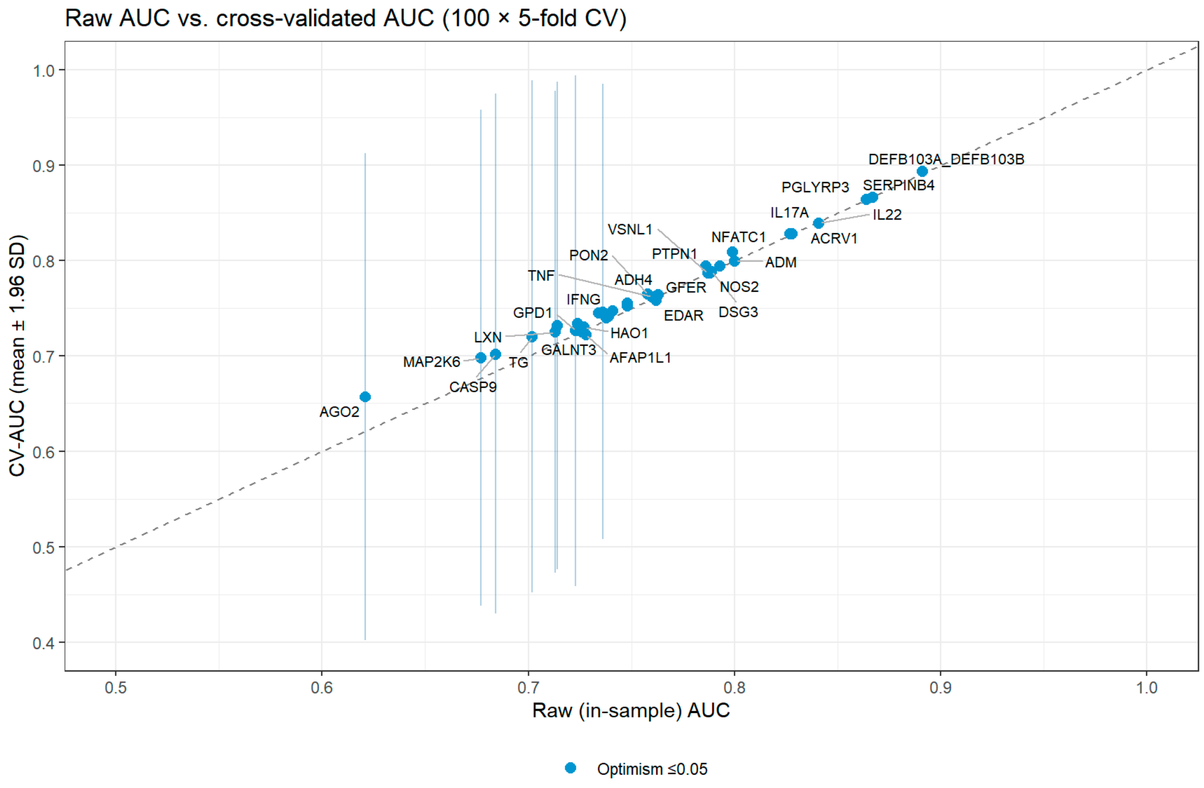

Raw AUC vs. CV-AUC for all 37 analytes. Dashed diagonal = no optimism. Points above diagonal are optimistic. Vertical bars show ±1.96 SD of CV-AUC distribution.
