# Supplementary material for: APOA1, DEFB103A_DEFB103B and DSG3 Are Novel Circulating Biomarkers of Psoriasis
Source: Int J Mol Sci. 2026 Jun 26;27(13):5805. doi: 10.3390/ijms27135805 (PMC13362322; doi:10.3390/ijms27135805)
Supplement: Supplementary file 1 [file ijms-27-05805-s001.zip › Supplementary Table S1_models.pdf]

Supplementary Table S1. Crude and adjusted logistic regression (Firth) for top 10 proteins by CV-AUC. Adjusted model controls for age, sex, and BMI (all standardised). BH-FDR applied per model.

Model	Protein	OR (95% CI)	p	FDR	n
Crude	DEFB103A_DEFB103B	26.05 (5.47–237.53)	1.00e-07	1.10e-06	60
	SERPINB4	6.46 (2.55–22.97)	2.00e-07	1.20e-06	60
	PGLYRP3	6.99 (2.7–24.82)	1.00e-06	3.40e-06	60
	IL17A	2.68 (1.64–5.18)	4.40e-06	1.09e-05	60
	ACRV1	2.82 (1.69–5.39)	1.01e-05	2.02e-05	60
	IL22	3.5 (1.84–8.06)	1.63e-05	2.72e-05	60
	ADM	0.68 (0.54–0.82)	2.76e-05	3.94e-05	60
	NFATC1	0.27 (0.12–0.54)	8.81e-05	1.10e-04	60
	PTPN1	0.54 (0.36–0.77)	2.81e-04	3.12e-04	60
	NOS2	5.68 (1.99–21.74)	5.28e-04	5.28e-04	60
Adjusted (age + sex)	DEFB103A_DEFB103B	17.59 (3.88–134.6)	2.23e-05	2.23e-04	56
	ADM	0.66 (0.48–0.83)	2.44e-04	1.10e-03	56
	IL17A	2.96 (1.53–7.52)	3.86e-04	1.10e-03	56
	SERPINB4	4.36 (1.71–16.14)	4.99e-04	1.10e-03	56
	IL22	3.09 (1.57–7.52)	5.78e-04	1.10e-03	56
	NFATC1	0.25 (0.09–0.57)	6.57e-04	1.10e-03	56
	PTPN1	0.51 (0.3–0.78)	1.13e-03	1.61e-03	56
	PGLYRP3	5.29 (1.77–22.13)	1.33e-03	1.67e-03	56

Model	Protein	OR (95% CI)	p	FDR	n
	ACRV1	2.48 (1.32–5.33)	3.72e-03	3.75e-03	56
	NOS2	5.34 (1.67–23.09)	3.75e-03	3.75e-03	56
