# Supplementary material for: APOA1, DEFB103A_DEFB103B and DSG3 Are Novel Circulating Biomarkers of Psoriasis
Source: Int J Mol Sci. 2026 Jun 26;27(13):5805. doi: 10.3390/ijms27135805 (PMC13362322; doi:10.3390/ijms27135805)
Supplement: Supplementary file 1 [file ijms-27-05805-s001.zip › Supplementary Table S3_miRNA.pdf]

Supplementary Table S3. Circulating miRNAs altered in Pso patients, reported as transported in plasma in complex with AGO2. The miRNAs were identified by comparing the list of miRNAs altered in Pso patients provided in publications cited in the table, with the list of miRNAs transported with AGO2 provided by Geekiyanage et al. (Dataset S2 in PMID: 32929008).

**miRNA name**	**Source of miRNA (Serum/plasma)**	**Compared groups**	**Ref**
miR-223	Plasma	PsO patients and healthy controls	PMID: 37700769
let-7b-3p<br>miR-29c-3p<br>miR-99a-5p<br>miR-140-5p<br>miR-181a-5p<br>miR-182-5p<br>miR-363-3p<br>miR-486-5p<br>miR-660-5p	Plasma	PsO patients before and after 1 year of treatment with risankizumab	PMID: 39800831
let-7e-5p<br>miR-15a-5p<br>miR-21-5p<br>miR-125b-5p<br>miR-141-3p<br>miR-181a-5p<br>miR-181b-5p	Serum	PsO patients and healthy controls	PMID: 36734535
